# A new *Plasmodium* Marchiafava & Celli, 1885 (Apicomplexa: Haemosporida) species in Cory’s Shearwater (*Calonectris borealis*) [Cory]) (Aves: Procellariiformes) stranded in a coastal area in Brazil

**DOI:** 10.1007/s11230-026-10281-z

**Published:** 2026-05-16

**Authors:** Carolina Clares dos Anjos, Bruna Antonetti Damato, Raquel Beneton Ferioli, Angélica M. Sánchez-Sarmiento, Amanda R. da Mata, Carla B. Barbosa, Hugo Gallo Neto, Carolina Romeiro Fernandes Chagas, Gediminas Valkiūnas, Karin Kirchgatter

**Affiliations:** 1 Programa de Pós-Graduação em Doenças Infecciosas e Saúde Global, Faculdade de Medicina, Universidade de São Paulo, São Paulo, SP 05403-000 Brazil; 2 Instituto Argonauta para a Conservação Costeira e Marinha, Ubatuba, SP 11695240 Brazil; 3 State Scientific Research Institute Nature Research Centre, Akademijos 2, 08412 Vilnius, Lithuania; 4 Instituto Pasteur, São Paulo, SP 01027-000 Brazil

## Abstract

Cory’s Shearwater (*Calonectris borealis*), a migratory seabird from the Procellariidae family, is widely distributed across the Atlantic Ocean. It breeds on islands of Azores, Madeira, Canary, and Berlengas, and winters in coastal areas of South America and Africa. This species’ wide habitat range contributes to pathogen exchange across global regions. *Plasmodium*, a genus of apicomplexan parasites causing avian malaria, affects many wild bird species worldwide. Although rare in seabirds, *Plasmodium* infections can significantly impact these hosts. We identified and characterized a new *Plasmodium* species in an adult male *C. borealis* found live-stranded on the northern coast of São Paulo, Brazil, during the daily activities of the Santos Basin Beach Monitoring Project (PMP-BS). The bird was rescued and kept in rehabilitation for 61 days but was euthanized due to poor prognosis. Blood smears revealed parasitemia varying from 0.04% until 2.09%. Blood and tissue samples were tested using PCR targeting a cytochrome b (*cytb*) gene fragment. The obtained sequence was 100% similar to *Plasmodium* lineage pLK06. Phylogenetic analysis placed the parasite within the *Novyella* clade, with morphological features closely related to *P. vaughani*. Necropsy showed hepato- and splenomegaly, while histopathology confirmed hepatitis and pulmonary hemorrhage. These findings, combined with clinical relapse despite anti-malaria treatment, indicate the parasite’s pathogenicity in *C. borealis*. This represents the first haemosporidian infection in this seabird and the first identification of the pLK06 lineage in Procellariiformes, resulting in the description of *Plasmodium borealis* **sp**. **nov**. These findings expand knowledge of parasite diversity in seabirds and highlight the need for continued surveillance in understudied avian groups.

**Supplementary Information:**

The online version contains supplementary material available at 10.1007/s11230-026-10281-z.

## Introduction

Cory's Shearwater (*Calonectris borealis*) is a migratory seabird from the family Procellariidae, widely distributed across the Atlantic Ocean, breeding in the North Atlantic on islands of Azores, Madeira, Canary, and Berlengas. During winter, they migrate to South Atlantic, particularly to the coastal areas of Brazil, Uruguay, and Argentina, but also in some parts of western Africa (Camphuysen & Van Der Meer, 2001; González-Solís et al., 2007; Somenzari et al., 2018; Pacheco et al., 2021; del Hoyo et al., 2024). Long-distance migrations of seabirds likely promote the global dispersal of pathogens (e.g., bacteria, viruses, eukaryotic parasites) in comparison to the non-migrating (resident) birds or short-distance migrants (Dietrich et al., 2011; McCoy et al., 2016; Boulinier et al., 2016), making the former ideal model organisms for studying pathogen distribution.

In general, long-distance migrant seabird species, such as Cory's shearwaters, appear resilient to certain parasites; however, their health is influenced by ecological pressures, including predation and pollution. In addition, plastic ingestion affects approximately 90% of seabirds, further compromising their health and serving as an environmental stressor that can influence parasite dynamics (Rodríguez et al., 2024).

Parasites of the genus *Plasmodium* (Apicomplexa: Haemosporida), transmitted by mosquitoes (Diptera: Culicidae), are the agents of avian, mammalian, and reptilian malaria (Garnham, 1966; Valkiūnas, 2005; Santiago-Alarcon et al., 2012). These parasites are a globally distributed group of heteroxenous protists affecting a wide range of wild birds (Clark et al., 2014). Almost 60 species of avian *Plasmodium* have been described so far, exhibiting considerable variation in geographic distribution, pathogenicity, and host specificity (Valkiūnas & Iezhova, 2018; Platonova et al., 2021; Vieira et al., 2025; Alves et al., 2026). The spread of *Plasmodium* to higher latitudes, such as the Arctic, due to climate change, poses a risk to naive bird populations. This expansion could lead to new outbreaks in regions previously free from avian malaria (Loiseau et al., 2012), as well as potential reports of different lineages in new hosts.

While the impact of these parasites on poultry, captive, and terrestrial birds is relatively well studied, research on seabirds remains limited (Khan et al., 2019). Most studies have been conducted in Europe and Africa, providing only weak evidence of infections by haemoparasites in this bird group (Quillfeldt et al., 2010; Hervías et al., 2013; Campioni et al., 2018). In Procellariiformes, few studies on haemosporidian species are available (Warner, 1968; Quillfeldt et al., 2010; Campioni et al., 2018; Vanstreels et al., 2020; Ilahiane et al., 2022; Bennett et al., 2024; Machado et al., 2025), and only *Plasmodium* parasites have been reported so far (Sgarioni et al., 2024). Moreover, no haemosporidian lineages have been associated with *C. borealis* in the MalAvi Database (currently available at https://tavimalara.shinyapps.io/malavi_tables/, accessed on 03/31/2025), highlighting the global gap of studies on this bird species. Overall, data on the prevalence of haemosporidian parasites in seabirds remain scarce (Warner, 1968; Quillfeldt et al., 2010; Vanstreels et al., 2014; Campioni et al., 2018; Kleinschmidt et al., 2022; Sgarioni et al., 2024).

Therefore, this study aims to molecularly and morphologically characterize a new *Plasmodium* species infecting a Cory’s Shearwater (*C. borealis)* found on the northern coast of São Paulo, Brazil, while presenting the clinicopathological findings associated with this infection. A phylogenetic reconstruction was performed to determine the evolutionary relationships of the new species with formerly described *Plasmodium* parasites.

## Materials and Methods

### Case presentation

**Fig. 1:**
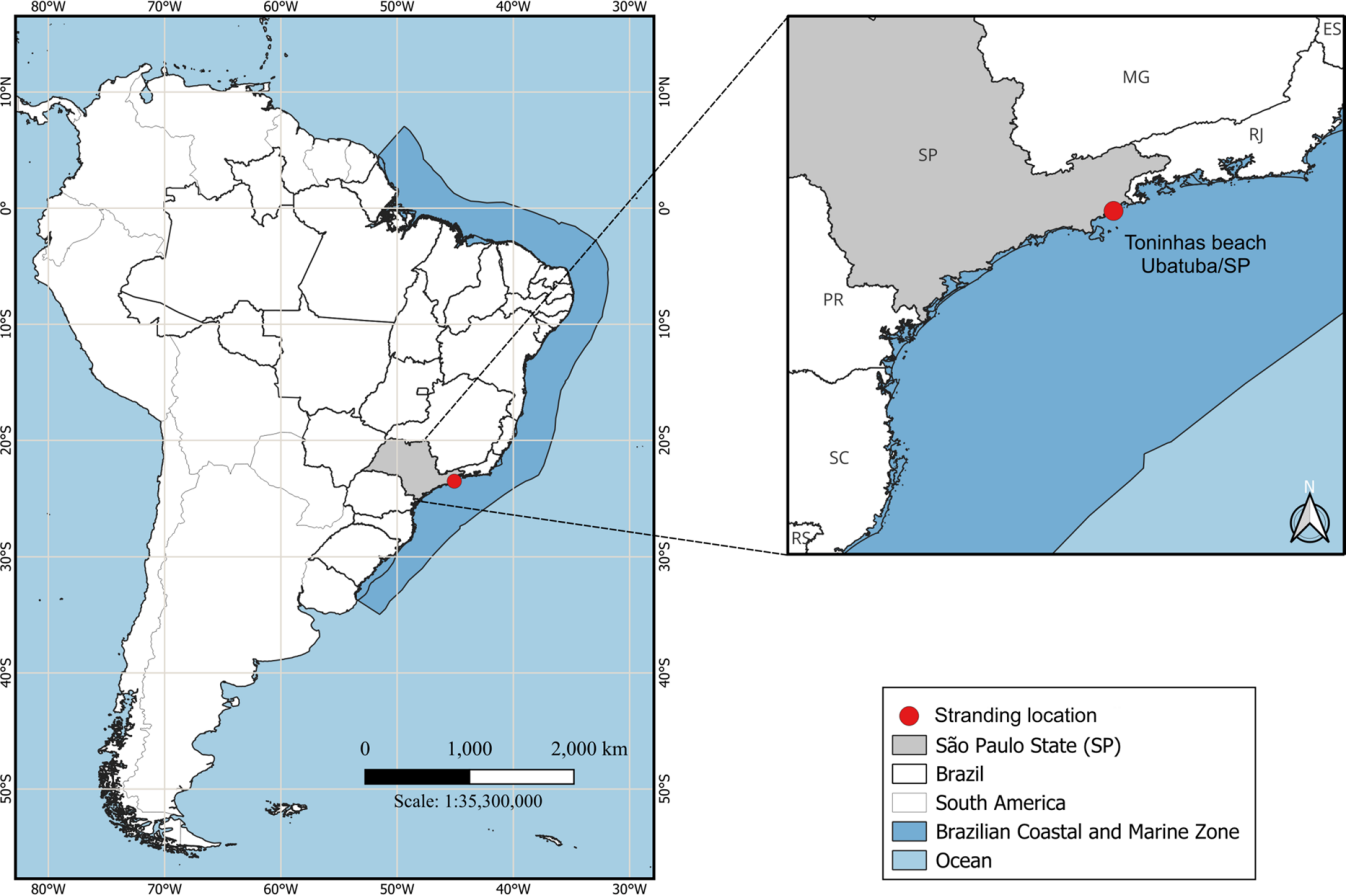
Stranding location of the *Calonectris borealis* specimen (IA16278) analyzed in this study.

On January 25, 2024, a Cory’s Shearwater (*Calonectris borealis*) individual (IA16278) was found in Toninhas Beach, Ubatuba, located on the northern coast of São Paulo (SP), Brazil (23°28.902′ S, 45°04.343′ W), during daily activities of the Santos Basin Beach Monitoring Project (PMP-BS) (Fig. 1). After an initial assessment ruled out clinical signs of avian influenza, the bird was transferred to the rehabilitation and de-oiling center of the Instituto Argonauta in Ubatuba, São Paulo, Brazil, for treatment.

Upon admission, the bird was in poor physical condition, with a body mass of 555 g, dehydrated, anemic, exhibiting a notable ectoparasite infestation, with poor waterproofing, and heavily worn plumage.

Comprehensive diagnostic evaluations were conducted to determine the underlying cause of the bird's condition. These included coproscopy (direct exam); hematology (hemogram and blood smears examination) following standard methodologies (Bried et al., 2011; Campbell & Grant, 2022); blood biochemistry (uric acid, albumin, aspartate aminotransferase – AST, alanine aminotransferase – ALT, calcium, phosphorus, creatine kinase – CK, gamma-glutamyl transferase – GGT, creatinine, lactate dehydrogenase – LDH, globulin, glucose, total proteins, alkaline phosphatase) using commercial kits and automated/semiautomated machines (Bioplus model Bio 200/2000) and radiographic exams.

Initial assessments indicated the presence of haemosporidian and coccidian infections. Samples were submitted for further morphological and molecular characterization, and medical treatment was initiated shortly after (see below). To prevent exposure to mosquitoes, the specimen was kept in a screened enclosure equipped with a fan. Insect repellent (diethyltoluamide-DEET, 6.65%) was applied to the bird (Vanstreels & Parsons, 2014; Hurtado et al., 2021) at least twice a day. Weekly physical examinations and blood sample collections were performed throughout the rehabilitation period to monitor health progression.

The bird initially showed signs of recovery; however, a relapse occurred. Due to its clinical condition worsening, the individual was euthanized after 61 days and submitted to necropsy on the same day.

### Sampling for haemosporidian parasites investigation

On admission, during clinical examinations (before and following treatment), and on necropsy, blood samples were collected by venipuncture from the jugular vein or intracardiac (necropsy) using a 22-24 gauge and 2-2.5 cm needle. Blood samples were transferred to collection tubes containing EDTA, and a few drops were placed into FTA® (Whatman®) blood cards. Blood smears were immediately prepared for microscopic analysis to detect blood parasites.

Primarily, as part of the admission exams protocols of the PMP-BS performed at the laboratory of the rehabilitation and de-oiling center of the Instituto Argonauta, blood smears were immediately fixed with absolute methanol and subsequently stained with a 10% Giemsa solution (Merck KGaA, Darmstadt, Germany) for 60 minutes (Valkiūnas, 2005). White blood cell differentials and hemoparasite assessments were conducted using optical microscopy (Campbell & Grant, 2022). Initial results indicated the presence of haemosporidian parasites, after which samples were submitted for morphological and molecular characterization at the Pasteur Institute.

The blood smears were analyzed at the Pasteur Institute under light microscopy using a ZEISS® Axio Lab.A1 microscope (ZEISS®, Jena, Germany). Diagnosis was based on the examination of 100 fields at 400 × magnification, followed by another 100 fields using an immersion objective (1000 × magnification). Parasites were identified based on available taxonomic keys (Valkiūnas, 2005; Valkiūnas et al., 2009; Mantilla et al., 2013; Walther et al., 2014; Valkiūnas & Iezhova, 2018). The intensity of parasitemia (%) was determined by counting the number of infected cells in 10,000 erythrocytes in randomly selected fields of a thin blood smear (Godfrey et al., 1987). Parasite images were captured using a ZEISS® Axiocam 305 color camera (ZEISS®, Jena, Germany) and processed using integrated software. Morphometric analysis was conducted manually using ImageJ software (Schneider et al., 2012), following the parameters outlined by Valkiūnas (2005). The nucleus displacement ratio (NDR) was calculated based on the method described (Bennett & Campbell, 1972). Statistical analyses were carried out using ‘R’ (version 4.3.3, 2024-02-29), package version dplyr (version 1.2.1.), inside R Studio (version "Kousa Dogwood", 2025-02-02).

During the necropsy, samples of spleen, brain, heart, liver, bone marrow, skeletal muscle, pancreas, lung, and kidney were collected in 10% neutral-buffered formalin for histopathological analysis. Additionally, small pieces of the mentioned organs were placed in separate tubes and frozen at − 20 °C for molecular analyses.

### Anti-malarial treatment

Based on the results obtained from the microscopical and molecular analysis of the samples collected during the admission, on February 1^st^ the treatment for avian malaria was initiated with hydroxychloroquine and addition of silymarin as a hepatoprotective (Vanstreels et al., 2014). The treatment protocol is described in Table 1.

**Table 1:** Drug treatment used in the *Calonectris borealis* (IA16278) analyzed in this study.

Drug	Dose (mg/kg)	Frequency	Treatment	
			Initiation	Duration (days)
Hydroxychloroquine	10	Q4H	Day 1	1
	5	SID	Day 2-10	9
Silymarin	100	BID	Day 1-20	20
Sulfamethoxazole-trimethoprim	40	BID	Day 11-20	10

Q4H: every four hours. SID: once a day. BID: twice a day.

### Molecular detection of parasites (DNA extraction, PCR, and sequencing)

DNA was extracted from the FTA cards using the commercial kit Wizard SV 96 Genomic DNA Purification System (PROMEGA®, Madison, Wisconsin, USA), following the manufacturer’s instructions. DNA from blood in EDTA and tissue samples was extracted using the Whole Blood Genomic DNA Extraction kit (Uniscience®) and the UNIXTRACTOR (Uniscience®) system, a semi-automated platform for nucleic acid extraction by magnetic beads, according to the manufacturer's instructions, with an additional wash step.

Next, the molecular diagnosis of haemosporidian parasites was performed using a PCR protocol targeting a ~500 bp fragment of the mitochondrial cytochrome *b* (*cytb*) gene. The first-round PCR was conducted using the primers HaemNFI and HaemNR3, with 2 μL (50 ng) of genomic DNA (gDNA). For the second-round nested PCR, 1 μL of the first PCR product per sample was used as a DNA template. Two primer sets were used to amplify *Plasmodium*/*Haemoproteus* (HaemF and HaemR2) and *Leucocytozoon* (HaemFL and HaemR2L) DNA (Bensch et al., 2000; Hellgren et al., 2004). In each PCR, positive controls were carried out in parallel and contained *Plasmodium* (*Novyella*) *nucleophilum* (lineage DENPET03), *Haemoproteus* (*Parahaemoproteus*) *nucleocentralis* (lineage TANDES01), or *Leucocytozoon* sp. (lineage ELAALB05) DNA, with ultrapure water served as a negative control.

Additionally, all samples that were positive in the first PCR were submitted to a second PCR protocol to amplify a longer *cytb* fragment (1131 bp). The first-round PCR was conducted using primers DW2 and DW4, with 2 µL (50 ng) of gDNA as a template. For the second-round nested reaction, 1 µL of the first-round PCR product was used as a template, and the reaction was carried out using primers DW1 and DW6. For sequencing, a pair of internal primers (DW3 and DW8) were also employed (Perkins & Schall, 2002).

Considering the coccidian positivity on admission, a PCR protocol targeting the parasites of class Conoidasida was also performed to amplify ~1035 bp of the ribosomal *18S* rRNA gene. The first PCR was conducted using primers Cocc18S_n1F and Cocc18S_n1R, with 2 μL (50 ng) of gDNA. For the nested PCR reaction, 1 μL of the first-round PCR product per sample was used as the DNA template. The reaction was carried out using primers Cocc18S_n2F and Cocc18S_n2R. For sequencing, the primers from the nested reaction were used (Chagas et al., 2021b). In each PCR, positive controls containing *Isospora* sp. or *Hepatozoon caimani* DNA were run in parallel, with ultrapure water serving as the negative control.

PCR amplification products were visualized by electrophoresis on a 1% agarose gel (Invitrogen®) using a molecular marker (1 Kb Plus DNA Ladder™, Invitrogen®) for 40 minutes at 90 V. DNA bands were marked with GelRed™ (Biotium®) and visualized under a UV transilluminator.

### Sequencing

Positive PCR products were subjected to sequencing using the Sanger method with the BigDye Terminator v3.1 Cycle Sequencing Kit (Applied Biosystems®, Carlsbad, CA, USA). Sequencing was performed using an ABI PRISM® 3500 Genetic Analyzer (Applied Biosystems, USA), a multi-user instrument available at the Institute of Tropical Medicine. The thermal cycling conditions included an initial step at 96 °C for 1 minute, followed by 30 cycles of 96 °C for 15 seconds, 50 °C for 15 seconds, and 60 °C for 4 minutes. Both strands were sequenced, and the sequences were corrected and aligned using the SeqMan software (DNASTAR Lasergene, version 7.0.0) to generate the consensus sequence. Parasite lineages were identified by aligning the consensus sequences with reference sequences using the BLAST (Basic Local Alignment Search Tool) algorithm in MalAvi (Bensch et al., 2009) and GenBank databases.

### Phylogenetic analysis

The phylogenetic reconstruction was performed using a set of haemosporidian *cytb* sequences from the MalAvi database (currently available at https://tavimalara.shinyapps.io/malavi_tables/, accessed April 11, 2024). We included 29 *Plasmodium* lineages deposited in GenBank, in addition to the sequence obtained in this study (GenBank PX712888, 479 bp). The tree was rooted using four lineages of the sister genus *Haemoproteus*, along with *Leucocytozoon* sp. lineage ISISKIN2. The alignment was performed using MEGA 12: Molecular Evolutionary Genetics Analysis program (Kumar et al., 2018). A Bayesian inference was conducted with MrBayes software v.3.2 (Ronquist et al., 2012), using a GTR+G+I model as suggested by the software MrModeltest 2.2 (Nylander, 2004) (software available from https://github.com/nylander/MrModeltest2). For the analysis, two Markov chains were run for 2 million generations. The first 25% of the trees were discarded as a burn-in, and the remaining trees were used to calculate posterior probabilities. The phylogenetic trees obtained from all analyses were visualized and edited using FigTree v. 1.4.0 (Rambaut, 2006) and Inkscape v1.3.2.

## Results

### Microscopic Examination and PCR results

Examination of blood smears collected from the *C. borealis* between January and March 2024 revealed the presence of intraerythrocytic parasites morphologically resembling *Plasmodium* spp. on five out of nine sampling dates. While in PCR analysis,  three out of nine samples were positive (see Table 2). On the admission hemogram, January 27^th^, blood smear analysis showed all blood stages of *Plasmodium* spp., with a parasitemia of 1.16%.

**Table 2:** Results obtained in the blood smear analysis and PCR of the Cory’s Shearwater *Calonectris borealis* (IA16278) in rehabilitation in 2024.

Date	Microscopy	Parasitemia	PCR result	Obs
Jan/27	*Plasmodium* sp.*	1.16%	Positive	Admission
Jan/31	*Plasmodium* sp.*	0.81%	NT	Treatment started on Feb/01
Feb/11	*Plasmodium* sp.	0.04%	Positive	–
Feb/21	Negative	–	NT	–
Feb/28	Negative	–	NT	–
Mar/05	Negative	–	NT	–
Mar/09	Negative	–	NT	–
Mar/25	*Plasmodium* sp.	2.09%	NT	Relapse of infection
Mar/26	*Plasmodium* sp.	1.32%	Positive	Euthanized/post-mortem**

(*) Presumptive (morphologic) parasite identification. (**) Blood coagulation was noted. NT: not tested.

Subsequent microscopic examination on February 11^th^ indicated a reduction in parasitemia to 0.04%. The parasites were identified as *Plasmodium* species, with genus confirmed by PCR. Follow-up examinations conducted on February 21^st^, February 28^th^, March 5^th^, and March 9^th^ revealed absence of parasites in the blood smears. On March 25^th^, a relapse was observed with a mean parasitemia of 2.09%. Euthanasia was performed on March 26^th^, and a blood smear was prepared post-mortem, indicating a lower parasitemia (1.32%). A summary of this data is presented in Table 2**.**

PCR for haemosporidian parasites confirmed the presence of *Plasmodium* sp., and the obtained sequence had 100% similarity with the lineage pLK06. The same sequence was obtained from blood in EDTA, blood in the FTA card, and all tested organs (spleen, brain, heart, liver, bone marrow, skeletal muscle, pancreas, lung, and kidney). Additionally, the PCR for coccidians also confirmed the presence of *Plasmodium* sp. from blood in EDTA, FTA card, and lungs. However, no lineage was attributed to this sequence, since it amplifies a fragment of the *18S* gene, and the barcoding region used for the hemosporidians is from the *cytb*. Blasting results showed that both *cytb* and *18S* sequences are closely related to *Plasmodium vaughani*, with a similarity of ~95%.

Morphological features of blood stages were the same in samples before and after treatment. While conducting a detailed morphological analysis of the blood smears prepared during the treatment, it was noticed that despite its close relation with *P. vaughani*, the parasite presented some different features that allowed us to describe a new *Plasmodium* species, attributing a morphospecies to the lineage pLK06.

**Description of** ***Plasmodium*** **(** ***Novyella*** **)** ***borealis*** **sp. nov.**

Taxonomic summary

Superphylum Alveolata Cavalier-Smith, 1991

Phylum Apicomplexa Levine, 1970

Subphylum Sporozoa Leuckart, 1879

Class Coccidea Leuckart, 1879

Order Haemosporida Danilewsky, 1885

Family Plasmodiidae Mesnil, 1903

Genus *Plasmodium* Marchiafava & Celli, 1885

Subgenus *Novyella* Corraetti, Garnham & Laird, 1963

**Type host**: Cory’s Shearwater *Calonectris borealis* (Cory) (Procellariiformes, Procellariidae).

**Type locality**: Toninhas beach, Ubatuba, São Paulo, SP (23°28.902′ S, 45°04.343′ W), Brazil.

**Type specimens**: Hapantotype (accession number IA16278, adult, male *Calonectris borealis*, parasitemia 2.09%, March 25^th^ 2024, Giemsa-stained blood films, Toninhas beach, Ubatuba, São Paulo, Brazil) was deposited at Instituto Argonauta para a Conservação Costeira e Marinha, Ubatuba, São Paulo, Brazil. Parahapantotype (accession numbers IA16278_1, IA16278_2, other data as for the hapantotype) was deposited in the Instituto Pasteur collection.

**Site of infection**: Mature erythrocytes; no other data.

**Prevalence**: Unknown. One examined Cory’s Shearwater (IA16278) was infected.

**Representative DNA sequence**: Mitochondrial *cytb* lineage pLK06 (479 bp, GenBank Accession Number PX712888; 1131 bp, GenBank Accession Number PZ273748); small subunit ribosomal RNA gene lineage pLK06 (830 bp, GenBank Accession Number PZ297969).

**Distribution**: The lineage pLK06 has been reported in Europe and Africa in birds belonging to ten families and 18 species, including both migratory and resident species of the orders Falconiformes and Passeriformes. Here, we report this lineage for the first time in South America, in *Calonectris borealis* (Procellariformes) (Table 3).

**Table 3:** Avian hosts and locations where the pLK06 lineage has been reported.

Order and Family	Host species	Site	References
**Falconiformes**			
Falconidae	*Falco naumanni*	Spain	Ortego et al. (2007) Ortego et al. (2008)
	*Falco eleonorae*	Spain, Canarias	Gutiérrez‑López et al. (2015)
**Passeriformes**			
Alaudidae	*Galerida cristata*	Morocco	Mata et al. (2015)
Fringillidae	*Fringilla coelebs*	Spain, Canarias	Bodawatta et al. (2020)
	*Emberiza cirlus*	Morocco	Mata et al. (2015)
Hirundinidae	*Delichon urbicum*	Spain	López-Calderón et al. (2019)
Motacillidae	*Anthus berthelotii*	Spain Spain, Canarias	Illera et al. (2008) and Spurgin et al. (2012) Bodawatta et al. (2020)
	*Anthus campestris*	Spain	Calero-Riestra and García (2016)
Muscicapidae	*Phoenicurus moussieri*	Morocco	Mata et al. (2015)
Paridae	*Cyanistes teneriffae*	Spain Spain, Canarias	Mata et al. (2015) Bodawatta et al. (2020)
	*Periparus ater*	Morocco	Mata et al. (2015)
Phylloscopidae	*Phylloscopus canariensis*	Spain, Canarias	Bodawatta et al. (2020)
Sylviidae	*Sylvia atricapilla*	Spain, Canarias	Bodawatta et al. (2020)
	*Curruca communis*	Sweden, Skane Lan	Ellis et al. (2020)
	*Curruca conspicillata*	Cape Verde Morocco Portugal, Madeira Spain, Canarias	Illera et al. (2015) Illera et al. (2015) and Bodawatta et al. (2020)
	*Sylvia melanocephala*	Spain, Canarias	Bodawatta et al. (2020)
Turdidae	*Erithacus rubecula*	Spain, Canarias	Bodawatta et al. (2020)
	*Turdus merula*	Spain, Canarias	Bodawatta et al. (2020)
**Procellariformes**			
Procellariidae	*Calonectris borealis*	Brazil, Ubatuba	This study

**ZooBank registration**: the life science identifier (LSID) for the new species *P. borealis* **sp**. **nov**. is urn:lsid:zoobank.org:pub:C1B4F877-3AD0-42D8-96F7-D20D7231BFE1.

**Vector**: Unknown.

**Etymology**: The species name refers to the host species *Calonectris borealis*, in which the parasite was discovered.

### Description

**Fig. 2:**
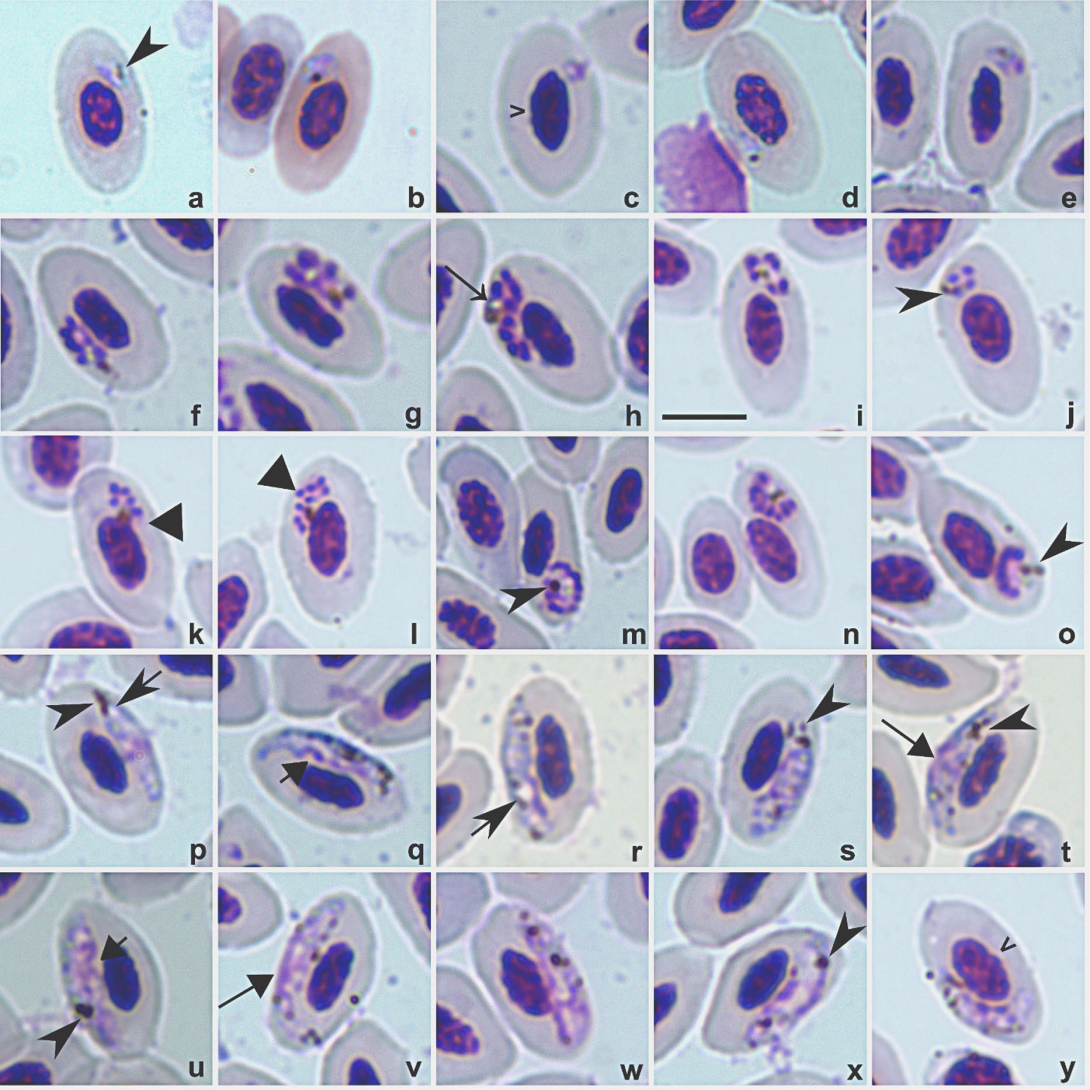
*Plasmodium* (*Novyella*) *borealis* **sp**. **nov**. (cytochrome *b* lineage pLK06) from the blood of *Calonectris borealis* (IA16278). Trophozoites (**a-e**), erythrocytic meronts (**f-o**), macrogametocytes (**p-t**) and microgametocytes (**u-y**). Note the presence of fan-like (**m-o**) meronts, refractive globules (**l**), scant (nearly invisible) cytoplasm in meronts (**h, j, k-o**) and the presence of vacuoles in macrogametocytes (**p-r**). Simple wide arrowhead: nuclei of infected erythrocytes; Triangle arrowhead: merozoites; Simple wide long arrow: refractive globules; Simple arrowhead: pigment granules; Short simple arrow: vacuole; Long triangle arrow: parasite nuclei; Short triangle arrow: unfilled space between gametocyte and erythrocyte nucleus. Methanol-fixed, Giemsa-stained thin blood smears. Scale bar: 10 µm. All images are from hapantotype.

**Trophozoites** (Fig. 2a-e) are seen in mature erythrocytes; present in variable shapes, but mostly even in outline (Fig. 2b, c); contain plentiful cytoplasm (Fig. 2b, d, e); usually in a polar or subpolar position in the infected erythrocytes (Fig. 2a-c, e), but occasionally in a lateral position (Fig. 2d); do not adhere to the nuclei of infected erythrocyte; predominantly possess one (Fig. 2b) or two (Fig. 2a) small pigment granules; the influence of trophozoites on infected erythrocytes is not pronounced.

**Erythrocytic meronts** (Fig. 2e-o) are seen only in mature erythrocytes; young meronts possess scant cytoplasm, which usually stains pale blue and usually is difficult to see in mature meronts (Fig. 2f-o); meronts usually lie free in the cytoplasm but some might slightly touch the nuclei of infected erythrocytes (Fig. 2 k, l, m); meronts can be seen anywhere in erythrocytes, including polar (Fig. 2j, o), subpolar (Fig. 2i, k-o), or lateral (Fig. 2f) positions in the infected cells; most of the fully grown meronts have a fan-like shape with peripheral location of merozoites (Fig. 2m-o), but some were irregular in form with randomly scattered merozoites (Fig. 2h, l); mature meronts contain four (Fig. 2j) to ten merozoites (Fig. 2m, o) (most frequently 5 and 7); meronts possess one or two medium-size (0.5 – 0.7µm) pigment granules (Fig. 2g, m), and sometimes two pigments of the same size were seen (Fig. 2n, o); pigment granules are usually closely appressed to the refractive globule (Fig. 2h, i, j, n); the influence of parasites on infected erythrocytes is not pronounced; fully grown meronts do not exceed 5.6 μm in length, and their size never exceeds that of the nuclei of infected erythrocytes (Table 4).

**Table 4:** Morphometry of erythrocytic meronts, gametocytes and host cells of *Plasmodium borealis* **sp**. **nov**. cytochrome *b* lineage pLK06 from the blood of *Calonectris borealis* (IA16278).

Feature	Range (Mean ± SD)
Uninfected erythrocyte (*n* = 31)	
Length	11.2-15.3 (13.4 ± 1.1)^a^
Width	6.4-8.5 (7.4 ± 0.5)
Area	59.1-94.4 (76.1 ± 9.5)
Uninfected erythrocyte nucleus (*n* = 31)	
Length	4.5-7 (6 ± 0.5)
Width	2.8-3.8 (3.3 ± 0.3)
Area	11.4-19.7 (15.4 ± 1.9)
Meront (*n* = 31)	
Length	2.6-5.6 (3.7 ± 0.8)
Width	1.6-3.4 (2.6 ± 0.5)
Area	5.5-10.8 (8.1 ± 1.5)
No. of merozoites (*n* = 31)	4-10 (6.4 ± 1.4)
No. of pigment granules (*n* = 31)	1-2 (1.2 ± 0.4)
Infected erythrocyte (*n* = 31)	
Length	8.2-15.4 (13.3 ± 2.1)
Width	4-8.3 (6.8 ± 1.1)
Area	27.7-92.3 (72.7 ± 18.4)
Infected erythrocyte nucleus (*n* = 31)	
Length	3-6.6 (5.4 ± 1)
Width	1.8-4.2 (3.2 ± 0.5)
Area	5.1-18.6 (13.6 ± 3.5)
Macrogametocytes (*n* = 18)	
Length	8.6-13.3 (10.9 ± 1.5)
Width	1.5-2.9 (2.2 ± 0.4)
Area	19-29.6 (24 ± 3.3)
No. of pigment granules (*n* = 16)	3-9 (5.9 ± 1.3)
Macrogametocytes nucleus (*n* = 18)	
Length	0.9-2.7 (1.8 ± 0.6)
Width	0.6-2.1 (1 ± 0.4)
Area	0.8-6.7 (3.1 ± 1.8)
NDR^b^	0.5-1.1 (0.8 ± 0.2)
Infected erythrocyte (*n* = 18)	
Length	11.1-15 (14 ± 0.9)
Width	6.8-8.5 (7.7 ± 0.5)
Area	73.4-89.4 (83.8 ± 4.9)
Infected erythrocyte nucleus (*n* = 18)	
Length	5.5-6.4 (5.9 ± 0.3)
Width	2.6-3.8 (3.2 ± 0.3)
Area	13.1-17.4 (14.9 ± 1.2)
Microgametocytes (*n* = 9)	
Length	9.9-14.3 (12 ± 1.7)
Width	1.5-2.7 (2.1 ± 0.4)
Area	21.5-29.3 (25.7 ± 2.8)
No. of pigment granules	7-15 (11.7 ± 2.4)
Microgametocyte nucleus^c^	–
NDR^b^	0.6-0.9 (0.7 ± 0.1)
Infected erythrocyte (*n* = 9)	
Length	12.8-15.4 (14.2 ± 0.9)
Width	6.7-8.4 (7.7 ± 0.6)
Area	76.9-98.8 (86.2 ± 7.1)
Infected erythrocyte nucleus (*n* = 9)	
Length	5.2-6.8 (6.1 ± 0.5)
Width	2.8-3.7 (3.4 ± 0.3)
Area	14.7-16.8 (15.7 ± 0.8)

^a^All measurements are given in micrometers (μm). Area given in µm^2^. Minimum and maximum values are provided, followed in parentheses by the arithmetic mean and standard deviation; ^b^NDR (Nucleus Displacement Ratio) according to Bennett and Campbell (1972); ^c^Microgametocyte nuclei were hardly defined and difficult to measure.

**Macrogametocytes** (Fig. 2p-t) are seen only in mature erythrocytes; the cytoplasm is granular in appearance (Fig. 2s, t); the outline is slightly irregular (Fig. 2q, r); young and mature gametocytes are elongated, seen in lateral position to the nuclei of infected erythrocytes and usually do not touch the host cell nuclei, but touch the envelope of the infected host cell (Fig. 2p-t); pigment granules vary from four to nine, roundish, medium-size (0.5 – 1.0 µm), scattered (Fig. 2q, r) but they may be clumped into a spot near the edge of the parasite (Fig. 2p, t); fully grown gametocytes exceed the size of the host cell nuclei, their size does not exceed 13.3 μm in length and less than 3 μm in width (Table 4); parasite nucleus is usually seen in a median and sometimes in a submedian position (Fig. 2p, t); a vacuole might be seen in gametocyte cytoplasm (Fig. 2p, r). The influence of gametocytes on infected erythrocytes is not pronounced.

**Microgametocytes** (Fig. 2u-y) present the general configuration and other features are as in macrogametocytes, with the usual haemosporidian sexual dimorphic characters; usually they possess more pigment granules scattered throughout the parasite cytoplasm (Table 4) in comparison to the macrogametocytes; can slightly deforms the nucleus of the infected host cell (Fig 2x, w) (Table 4).

### Remarks

Among avian malaria parasites, blood stages of *P. borealis* **sp**. **nov**. pLK06 are most similar to *P. vaughani* pSYAT05, both of which belong to the *Novyella* subgenus, with a genetic distance of 5% in the partial *cytb* gene. *Plasmodium borealis* **sp**. **nov**. pLK06 can be readily distinguished due to common mature erythrocytic meronts, in which merozoites assume fan-like position and contain 9-10 merozoites (Fig. 2, m-o); such meronts are absent in *P. vaughani*. Additionally, *P. borealis* **sp**. **nov**. pLK06 contains four to ten (most frequently 5 and 7) merozoites, while *P. vaughani* pSYAT05 produces four to eight (most frequently six) merozoites. Regarding the pigment granules in mature meronts, they are of medium-size (between 0.5 – 0.7 µm) in the new species exhibiting one (Fig. 2i, l, m) but sometimes two (Fig. 2g, o), and when two granules are present, they are of approximately the same size. In contrast, mature meronts of *P. vaughani* pSYAT05 can have from one to three small pigment granules (<0.5 µm), which are consistently of different size. The macrogametocytes’ cytoplasm of *P. borealis* **sp**. **nov**. pLK06 is granular in appearance (Fig. 2s, t), whereas in *P. vaughani* pSYAT05, it is predominantly homogeneous. *Plasmodium borealis* **sp**. **nov**. pLK06 has four to ten pigment granules in macrogametocytes; the number of pigment granules in *P. vaughani* pSYAT05 is more variable, and might vary between two to 24 (Valkiūnas, 2005; Zehtindjiev et al., 2012; Valkiūnas & Iezhova, 2018).

*Plasmodium borealis* **sp**. **nov**. pLK06 also should be distinguished from several other *Novyella* species, which are common in the Americas, have similar blood stages and are genetically closely related to it. These are *Plasmodium homopolare* pBAEBIC02*, Plasmodium unalis* pTFUS06*, Plasmodium hexamerium* pALEDIA02*,* and *Plasmodium parahexamerium* pALDI1*.* The following characters of blood stages are particularly helpful during these parasite species identifications.

Advanced and mature meronts of *P. homopolare* pBAEBIC02 assume precisely polar or subpolar positions in infected erythrocytes, while in *P. borealis* **sp**. **nov**. pLK06 they usually are seen anywhere in the infected cell, including polar (Fig. 2h, o), subpolar (Fig. 2j, k, m-o), or lateral (Fig. 2f) positions. Fully grown gametocytes of *P. homopolare* are attached both to the nuclei and envelope of erythrocytes, and they fill up poles of erythrocytes (Walther et al., 2014); these characters are not characteristic of *P. borealis* **sp**. **nov**. pLK06. Genetic distance between these parasite lineages is 4.2%.

*Plasmodium borealis* **sp**. **nov**. pLK06 shows markedly variable location of merozoites in mature meronts, whereas in *P. unalis* pTFUS06 the merozoites assume predominantly fan-like locations in the mature meronts. Furthermore, advanced and mature meronts of *P. borealis* **sp**. **nov**. often contain two pigment granules (Fig. 2g, o), but only one pigment granule is present in corresponding blood stages of *P. unalis* (Mantilla et al., 2013). Genetic distance between these parasite lineages is 5%.

*Plasmodium borealis* **sp**. **nov**. pLK06 can be readily distinguished from both *P. parahexamerium* pALEDIA02 (genetic distance between lineages is of 4%) and *P. hexamerium* pALDI1 (genetic distance of 4.1%) due to the number of merozoites, which are produced in erythrocytic meronts. Mature meronts of *P. hexamerium* and *P. parahexamerium* produce relatively stable number of merozoites (Huff, 1935; Valkiūnas et al., 2009), with over 90% of mature meronts containing six merozoites, while in the new species, the number of merozoites in mature meronts is markedly variable (varies from four to ten, most frequently is five and seven) (Fig. 2e-o).

### Phylogenetic Analysis

**Fig. 3:**
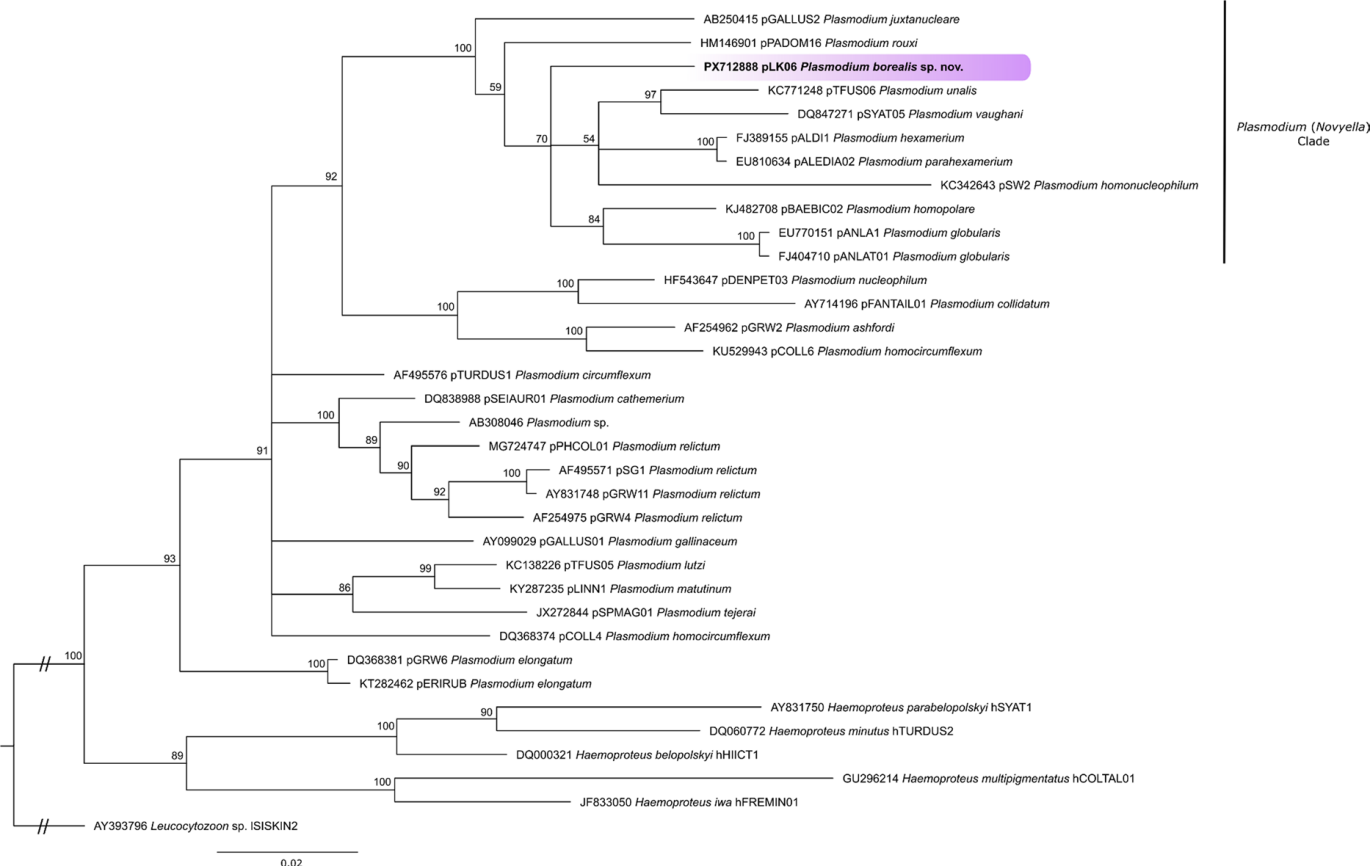
Phylogenetic tree inferred by Bayesian analyses based on 479 bp of the mitochondrial gene cytochrome *b* (*cytb*) of representative avian haemosporidian species. The sequence obtained in the present study is highlighted in bold and purple. Five lineages of *Haemoproteus* and one lineage of *Leucocytozoon* were used as outgroups. The support values of the nodes (in percentage) indicate posterior probabilities.

The genetic analysis showed that *P. borealis* **sp**. **nov**. is closely related to *P. hexamerium* pALDI1 (genetic distance of 4.1%)*, P. parahexamerim* pALEDIA02 (4%), *P. unalis* pTFUS06 (5%), *P. vaughani* pSYAT05 (5%), and *P. homopolare* pBAEBIC02 (4.2%) in partial *cytb* gene. However, morphological features of blood stages of the new species are most similar to *P. vaughani.* In the phylogenetic inference, all these parasites cluster in a well-supported clade with other *Plasmodium* (*Novyella*) species, with the new species being placed in a separate branch (Fig. 3).

The lineage pLK06 was reported in Falconiformes and Passeriformes birds in Africa and Europe; these belong to the ten families (Falconidae, Alaudidae, Fringillidae, Hirundinidae, Motacillidae, Muscicapidae, Paridae, Phylloscopidae, Sylviidae, and Turdidae) and 18 bird species (Table 3). Among the most often reported hosts are the African blue tit *Cyanistes teneriffae*, spectacled warbler *Curruca conspicillata*, and Berthelot's pipit *Anthus berthelotii*, all resident species in Africa and Europe (MalAvi Database, currently available at https://tavimalara.shinyapps.io/malavi_tables/, accessed on 07/11/2025).

### Coproscopy, hematology, and blood biochemistry results

Fecal examination performed on the first three days after admission (pooled samples from January 25^th^ till 27^th^) revealed a moderate parasitic burden (protozoan oocysts similar to enterococcidian parasites). Admission hemogram (January 27^th^) revealed hypochromic microcytic anemia with marked polychromasia and anisocytosis, as well as moderate parasitic burden of erythrocytic hemoparasites in blood smear (confirmed later as *Plasmodium* spp. using PCR, see Table 2). Biochemistry findings revealed a mild increase of AST (382 U/L), and mild hypoalbuminemia (0.7 g/dL) (Table 5). Control fecal examination (pooled samples from February 06^th^ until 08^th^) performed after antiparasitic treatment (Toltrazuril 5%, 7mg/kg, VI, SID, for 3 days) was negative.

**Table 5:** Hematological and serum biochemistry parameters of the *Calonectris borealis* (IA16278) through the rehabilitation period in 2024.

	Jan/27	Feb/01	Feb/11	Feb/21	Feb/28	Mar/09	Mar/23
**Hematological parameters**							
RBC (x10^6^/µL)	1.42	–	3.14	2.87	2.84	–	3.95
Hemoglobin (g/dL)	4.5	–	9.3	8.6	9.2	–	12.1
Hematocrit (%)	18	–	36	38	41	–	44
MCV (fL)	126.76	–	114.64	132.4	144.36	–	111.39
MCH (pg)	31.69	–	29.61	29.96	32.39	–	30.63
MCHC (%)	25	–	25.83	22.63	22.43	–	27.5
Total protein (g/dL)	3.2	–	5.4	5.2	4.4	–	5.5
WBC (x10^3^/µL)	4070	–	14630	4730	6368	–	6490
Heterophils (x10^3^/µL)	3093.2	–	9363.2	2601.5	3317.6	–	3634
Heterophils (%)	76	–	64	55	52	–	56
Eosinophils (x10^3^/µL)	122.1	–	1463	520.3	319	–	195
Eosinophils (%)	3	–	10	11	5	–	3
Basophils (x10^3^/µL)	0	–	0	0	0	–	65
Basophils (%)	0	–	0	0	0	–	1
Lymphocytes (x10^3^/µL)	854.7	–	2926	1608.2	2552	–	2142
Lymphocytes (%)	21	–	20	34	40	–	33
Monocytes (x10^3^/µL)	0	–	877.8	0	191.4	–	454
Monocytes (%)	0	–	6	0	3	–	7
**Serum biochemistry**							
Uric acid (mg/dL)	–	3.7	3.9	4	2.3	3	3.9
Protein (g/dL)	–	4.2	5.4	5.2	4.4	3.7	5.5
Albumin (g/dL)	–	0.7	1	1.2	NPI	1.3	1.1
Globulin (g/dL)	–	3.5	4.4	4	NPA	2.4	4.4
Calcium (mg/dL)	–	9.5	9.8.	11.5	10.4	11.9	NPI
Phosphorus (mg/dL)	–	3.5	1.8	2	3.3	2.1	NPI
Calcium to Phosphorus ratio	–	2.7	5.4	5.75	3.15	5.6	NPI
AST (U/L)	–	382	648	335	424	261	251
CK (U/L)	–	922	121	NPI	NPI	97	340
LDH (U/L)	–	NPI	NPI	NPI	307	226	NPI
Glucose (mg/dL)	–	259	183	204	178	156	138

(-) not performed. NPI = Not performed due to insufficient sample volume. NPA= Not performed due to no albumin determination.

On February 11^th^, the anemia persisted, a moderate increase of AST (648 U/L), an increased calcium to phosphorus relation (5.4), hypophosphatemia (1.8 mg/dL), and marked leukocytosis (14630 U/L) with monocytosis (877.8 U/L) were reported (Table 5). On February 21^st^, blood analyses indicated persistent anemia. AST decreased but persisted mildly high (335 U/L), the high calcium to phosphorus ratio persisted (5.7) (Table 5), and the anisocytosis became mild. On February 28^th^, moderate polychromasia decreased but still abnormal, calcium to phosphorus ratio (3.2), high LDH (307 U/L), decreased uric acid (2.3 mg/dL), increased AST (424 U/L) and hypoglycemia (178 g/dL) were noted (Table 5). On March 25^th^ (the day before euthanasia), the blood smear analyses revealed a parasitemia of 2.09%, characterizing malaria recurrence despite treatment. Kidney (uric acid – 3.9 mg/dL) and liver enzymes (AST – 251 U/L) were still abnormal (Table 5).

### Radiographic examination

X-rays performed on February 16^th^ revealed increased radiopacity of both kidneys, increased alveolar pattern and clavicular air sac radiopacity (suggesting an infectious process). Control X-rays on February 28^th^ showed a mild improvement on the air sacs; however, the alveolar pattern persisted. A significant increase in kidney radiopacity and hepatomegaly was noted.

### Gross pathology and histopathology findings

**Fig. 4:**
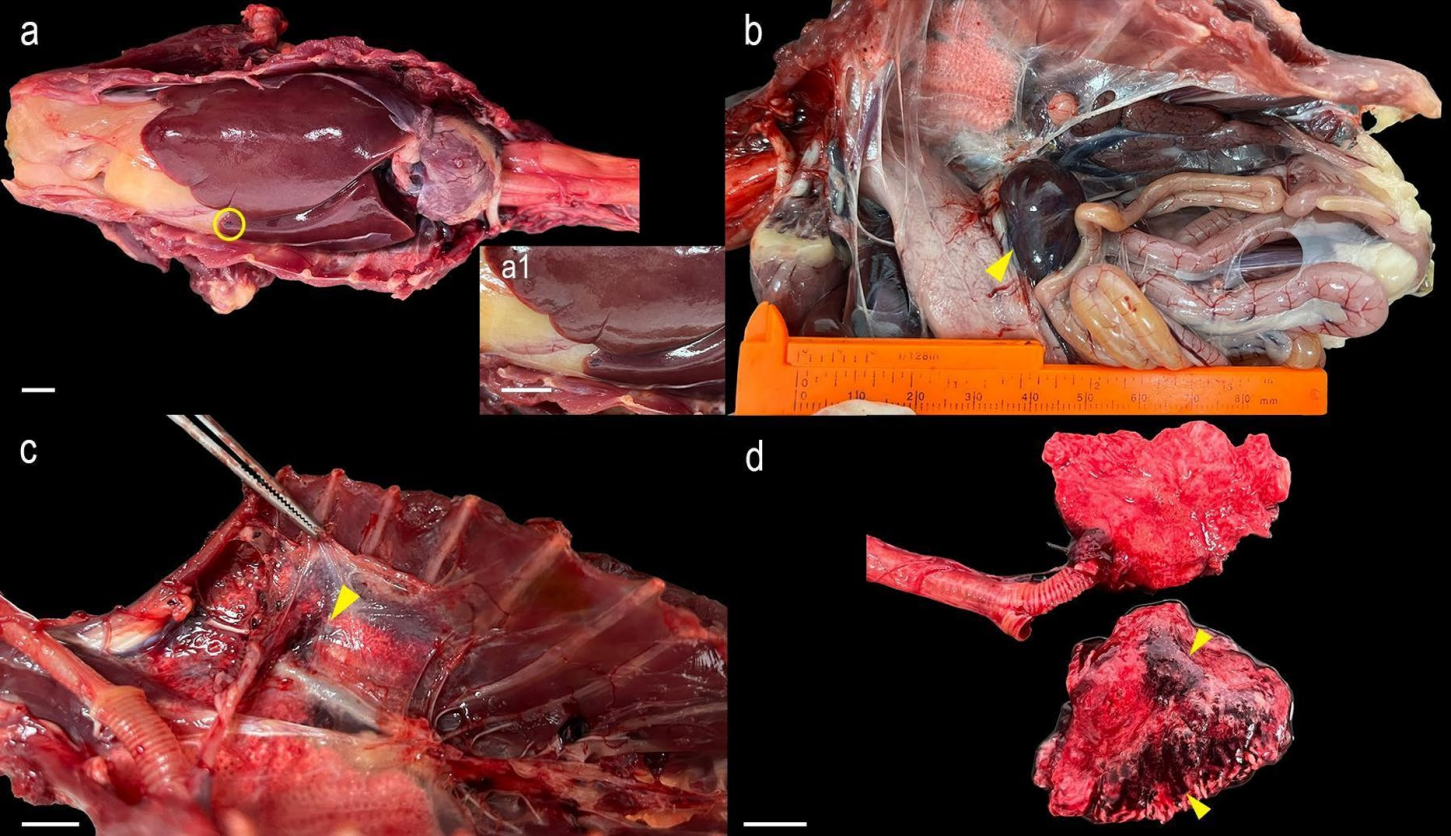
Gross findings of a Cory’s Shearwater (*Calonectris borealis*) (IA16278) positive for *Plasmodium borealis* **sp**. **nov**. (a) Mild to moderate diffuse hepatomegaly. Focal restricted hemorrhage in the apical edge of the left lobe (yellow circle); (a1) Insert, mild focal hemorrhage in the liver, with approximately 0.3 cm x 0.4 cm in diameter; (b) Celomic cavity, viscera *in situ*. Note diffuse severe splenomegaly (yellow arrowhead); (c) Ventral view, lungs *in situ*, mild to moderate multifocal pulmonary hemorrhage (yellow arrowhead); (d) Dorsal view of the lungs, extensive areas of multifocal hemorrhage (yellow arrowheads). Scale bars = 1 cm.

Grossly, diffuse severe splenomegaly, mild to moderate diffuse hepatomegaly, a focal hemorrhage in the liver, and pulmonary hemorrhage were noted (Fig. 4a). Two yellowish lesions of about 0.2 cm each were noted on the oropharynx mucosa (palate). A considerable amount of adipose tissue was observed in the coelomic cavity (body mass 785g) (Fig. 4b). In the ventral view (Fig. 4c), lungs *in situ* exhibited mild to moderate multifocal pulmonary hemorrhage, while the dorsal view revealed extensive areas of multifocal hemorrhage (Fig. 4d). Histopathology revealed chronic hepatitis characterized by moderate multifocal lymphocytic infiltration; marked peri bronchial focal hemorrhage and moderate multifocal congestion, and mild focal inflammatory heterophilic infiltrate and sero cellular crust associated with ulceration and mild presence of bacteria in the oropharynx. The presence of developed seminiferous tubules with multicellular walls and a lumen containing spermatozoids confirmed the individual as sexually mature. No lesions were observed in the cloaca, heart, brain, large and small intestine, muscle, thyroid and parathyroid, skin, thymus, pancreas, kidneys, and proventriculus.

## Discussion

*Plasmodium* infections in seabirds, including Procellariiformes, are rare yet can profoundly impact host health and population dynamics. The clinicopathological findings in this specimen mirror those reported previously (Vanstreels et al., 2014, 2015), with avian malaria often impairing behavior and physiology, manifesting as lethargy, anorexia, poor productivity, growth retardation, green feces, dyspnea, and sometimes death (Sorci & Møller, 1997; Atkinson, 2008; Schoenle et al., 2017). Although, in some cases, birds remain asymptomatic, severe infections may involve hepatomegaly with necrosis, fatty liver, splenomegaly, pericardial effusion, nephritis, pulmonary edema, brain capillary blockage, or hemorrhagic conjunctivitis, though symptom severity varies widely by host-parasite combination and infection stage, from mild signs to high mortality (Williams, 2005; Dinhopl et al., 2015; Ilgūnas et al., 2016; Atkinson et al., 1995; Atkinson, 2008; Palinauskas et al., 2008, 2011; Howe et al., 2012; Vanstreels et al., 2014, 2015).

In this study, the infected *C. borealis* showed improvements in nutritional status and anemia during rehabilitation, despite intermittent weight fluctuations, dyspnea episodes, selective appetite, and poor waterproofing. However, malaria recurred despite treatment, leading to euthanasia due to its fragile condition and poor prognosis, with renal and hepatic sequelae evident on X-rays, hematology, and biochemistry. Necropsy confirmed hepatomegaly, splenomegaly, and hemorrhagic-inflammatory lesions typical of chronic infection.

Upon admission to the rehabilitation center, the studied individual exhibited relatively high parasitemia (1.16%), which declined over the next few days (0.81%), continued decreasing after treatment initiation (0.04%), and eventually became undetectable for nearly a month. However, 34 days post-treatment, a relapse occurred with elevated parasitemia (2.09%). Reports on avian malaria treatment remain scarce, and success rates are controversial; even combined chloroquine (targeting erythrocytic meronts) and primaquine (addressing exoerythrocytic stages responsible for relapses) do not guarantee cure, with mortality often remaining high (Stoskopf & Beier, 1979; Remple, 2004; Bueno et al., 2010; Vanstreels and Parsons, 2014; Vanstreels et al., 2019).

Notably, the bird in this study received only chloroquine, as primaquine is restricted to human malaria treatment in Brazil, making relapse predictable (Vanstreels et al., 2014). The lower parasitemia (1.32%) in the *post-mortem* blood smear likely stemmed from poor sample quality due to increased blood viscosity and clotting after death. Indeed, *post-mortem* smears are unsuitable for detailed parasite morphology analysis, as death-induced changes distort parasite morphology and infection intensity.

Blood films were collected before and after treatment. Studies on human and rodent malaria report gametocyte morphological changes within hours of drug administration (Sachanonta et al., 2011; Dechy-Cabaret & Benoit-Vical, 2012), and similar effects have been suggested for avian malaria over the same timeframe (Vanstreels et al., 2014, 2019). Yet, no detailed avian studies exist, with prior reports limited to short-term drug effects (hours to one day post-administration). In the present study, morphology was assessed over a month post-treatment; no differences in blood-stage morphology were observed before and after using this protocol (see Fig. 2 and Supplementary Fig. 1).

All PCRs targeting the *cytb* haemosporidian barcoding fragment conducted in the blood samples and organs were positive for *Plasmodium* sp. pLK06, which is closely related to *P. vaughani* pSYAT05. The sequence obtained with the coccidian primers targeting the *18S* gene also provided a sequence closely related to *P. vaughani* pSYAT05. Interestingly, these primers were designed to target avian *Lankesterella* and related parasites (Chagas et al., 2021b). This protocol has been extensively used to diagnose avian *Lankesterella* and *Isospora* parasites, and it has never been reported to amplify *Plasmodium* or any other haemosporidian DNA (Chagas et al., 2021a, b; Keckeisen et al., 2024). This highlights the importance of conducting sequencing and not relying exclusively on PCR amplifications. Yet, since co-infections with multiple parasites are relatively common in the wild, it also shows that researchers targeting avian *Lankesterella* should bear in mind that haemosporidians can also be amplified, although probably rarely.

Seabirds such as common terns (*Sterna hirundo*) and Cory's shearwaters, exhibit exceptionally low prevalence of haemosporidian parasites, driven by ecological and biological factors (Khan et al., 2019). For example, common terns showed just 3.6% prevalence (Capasso et al., 2023), while Cory's shearwaters had none (Campioni et al., 2018). This pattern likely stems from their unique marine niches, which lack suitable vectors, as seen in the general scarcity of *Plasmodium* in oceanic habitats (Warner, 1968; Valkiūnas, 2005; Merino et al., 2012; Bastien et al., 2014; Campioni et al., 2018; Vanstreels et al., 2020), though seabirds remain underrepresented in haemosporidian surveys dominated by terrestrial and passerine species (Quillfeldt et al., 2010; Sgarioni et al., 2024).

*Plasmodium* impacts on seabirds can be severe, especially in naive populations (Gulliver et al., 2022), yet broader factors like climate change, habitat loss, and invasive species introductions heighten transmission risks, underscoring the need for vigilant monitoring (Vanstreels et al., 2014). Moreover, the low host specificity of *Plasmodium*, allowing infections across distantly related birds, undermines host identity as a reliable taxonomic trait and complicates species identification in unexpected wildlife hosts (Garnham, 1966, 1980; Valkiūnas & Ashford, 2002; Valkiūnas & Iezhova, 2018).

In this study, we report the first documented case of haemosporidian infection in *Calonectris borealis*. The combined morphological and molecular analysis supported the description of *Plasmodium* (*Novyella*) *borealis* **sp**. **nov**. pLK06, being also the first report of this parasite lineage in Brazil. The broad geographic distribution and migratory behavior of *C. borealis* facilitate the exchange of pathogens across regions, underscoring the importance of investigating underreported infectious agents in this bird group. The lineage pLK06 has been found in Europe (Spain, Portugal, and Sweden) and Africa (Morocco and Cape Verde), infecting Passeriformes and Falconiformes birds, which makes this the first report of this lineage in Brazil.

Regarding the records of avian malaria infection in Procellariformes, only a few of them are mentioned in the literature: one of *Plasmodium circumflexum* (pSW5) reported in Streaked Shearwater *Calonectris leucomelas*, in Japan (Inumaru et al., 2017); one of *Plasmodium* sp. in Galapagos Petrel *Pterodroma phaeopygia* in Hawaii, USA (Warner, 1968); one of *Plasmodium cathemerium* (pPADOM09) in Manx shearwaters *Puffinus puffinus*, in Brazil (Vanstreels et al., 2020); one of *Plasmodium relictum* (pSGS1) in slender-billed prion *Pachyptila belcheri* in the Falkland Islands, UK (Quillfeldt et al., 2010), and one of *Plasmodium matutinum* (pLINN1) in westland petrel *Procellaria westlandica* in New Zealand (Bennett et al., 2024).

In conclusion, this study expands the knowledge regarding *Plasmodium* occurrence in seabirds, with the Cory´s Shearwater *Calonectris borealis* as a new host for the pLK06 lineage. A new malaria parasite species description is presented: *Plasmodium borealis* **sp**. **nov**., along with the clinical and pathological findings associated with the infection in this host. The distinct morphological characteristics and genetic divergence in relation to closely related morphospecies (~5% in *cytb*) corroborate the recognition of *P. borealis* as a new species, although species delimitation within this group of parasites remains a challenge (Outlaw and Ricklefs, 2014). Understanding the pathogenic effects of this parasite is essential for assessing its influence on migratory bird populations, underscoring the importance of additional studies on its occurrence and consequences in avian hosts.

## Supplementary Information

Below is the link to the electronic supplementary material.

Supplementary file1 (DOCX 7959 kb)

## Data Availability

The datasets generated during the current study are available in the GenBank database (https://www.ncbi.nlm.nih.gov/nucleotide/) under accession numbers PX712888, PZ273748 and PZ297969, and in the MalAvi database.
