# Supplementary material for: A new Plasmodium Marchiafava & Celli, 1885 (Apicomplexa: Haemosporida) species in Cory’s Shearwater (Calonectris borealis) [Cory]) (Aves: Procellariiformes) stranded in a coastal area in Brazil
Source: Syst Parasitol. 2026 May 16;103(4):24. doi: 10.1007/s11230-026-10281-z (PMC13179919; doi:10.1007/s11230-026-10281-z)
Supplement: Supplementary file 1 — Supplementary file1 (DOCX 7959 kb) [file 11230_2026_10281_MOESM1_ESM.docx]

**Supplementary File**


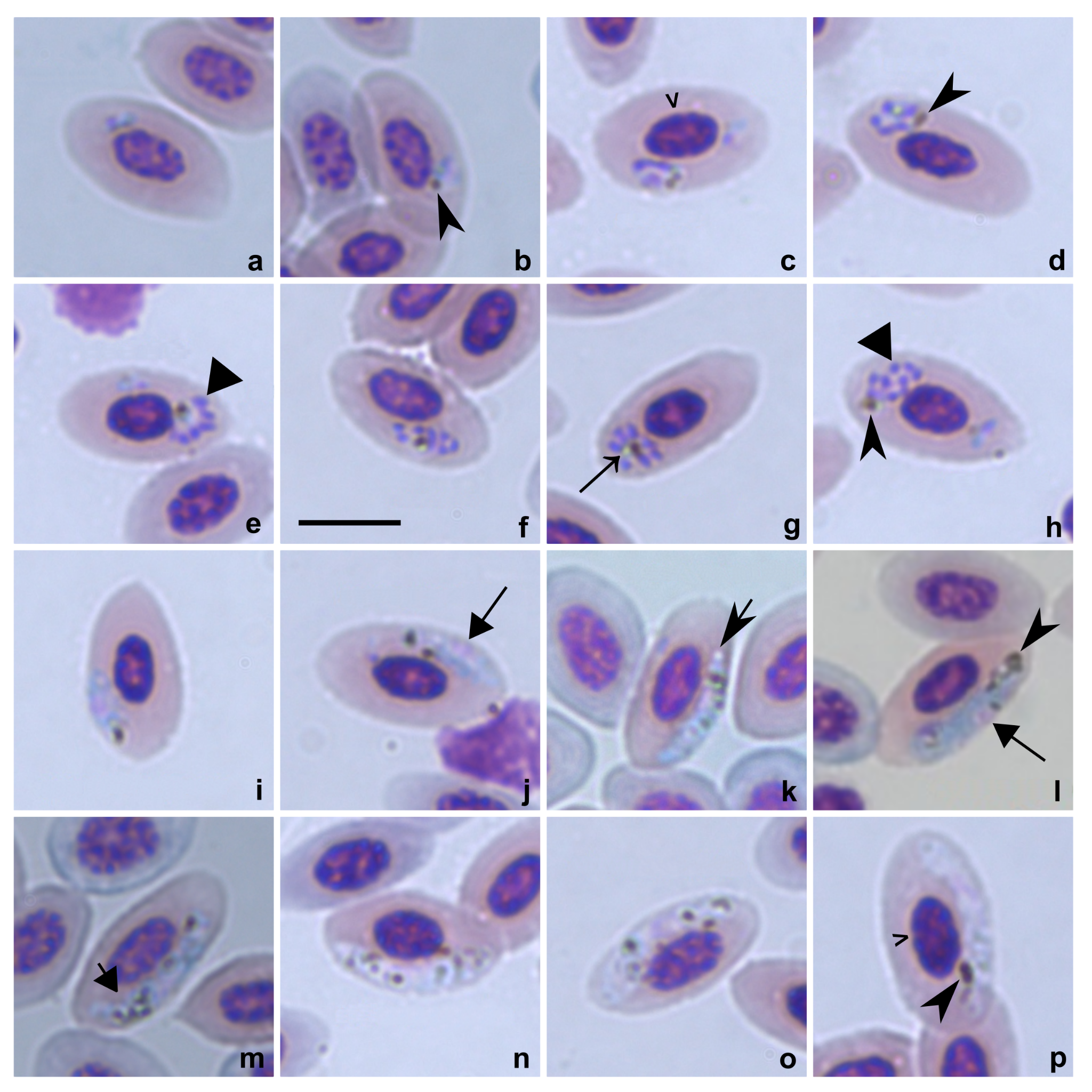


**Supplementary Figure 1:** *Plasmodium (Novyella) borealis* **sp. nov**. (cytochrome *b* lineage pLK06) from the blood of *Calonectris borealis* (IA16278) before treatment. Trophozoites (**a-b**), erythrocytic meronts (**c-h**), macrogametocytes (**i-l**) and microgametocytes (**m-p**). Note the presence of fan-like (**e**) meronts, refractive globules (**g**), scanty (nearly invisible) cytoplasm in meronts (**f-h**) and the presence of vacuoles in macrogametocytes (**k**). Simple wide arrowhead: nuclei of infected erythrocytes; Triangle arrowhead: merozoites; Simple wide long arrow: refractive globules; Simple arrowhead: pigment granules; Short simple arrow: vacuole; Long triangle arrow: parasite nuclei; Short triangle arrow: unfilled space between gametocyte and erythrocyte nucleus. Methanol-fixed, Giemsa-stained thin blood smears. Scale bar: 10 µm. All images are from hapantotype.
